# Quantitative Proteomics Indicate Radical Removal of Non-Small Cell Lung Cancer and Predict Outcome

**DOI:** 10.3390/biomedicines10112738

**Published:** 2022-10-28

**Authors:** Embla Bodén, Jesper Andreasson, Gabriel Hirdman, Malin Malmsjö, Sandra Lindstedt

**Affiliations:** 1 Department of Clinical Sciences, Lund University, 22362 Lund, Sweden; 2 Wallenberg Center for Molecular Medicine, Lund University, 22363 Lund, Sweden; 3 Lund Stem Cell Center, Lund University, 22362 Lund, Sweden; 4 Department of Cardiothoracic Surgery and Transplantation, Skåne University Hospital, 22242 Lund, Sweden

**Keywords:** non-small cell lung cancer, biomarkers, proteomics

## Abstract

Non-small cell lung cancer (NSCLC) is associated with low survival rates, often due to late diagnosis and lack of personalized medicine. Diagnosing and monitoring NSCLC using blood samples has lately gained interest due to its less invasive nature. In the present study, plasma was collected at three timepoints and analyzed using proximity extension assay technology and quantitative real-time polymerase chain reaction in patients with primary NSCLC stages IA–IIIA undergoing surgery. Results were adjusted for patient demographics, tumor, node, metastasis (TNM) stage, and multiple testing. Major histocompatibility (MHC) class 1 polypeptide-related sequence A/B (MIC-A/B) and tumor necrosis factor ligand superfamily member 6 (FASLG) were significantly increased post-surgery, suggesting radical removal of cancerous cells. Levels of hepatocyte growth factor (HGF) initially increased postoperatively but were later lowered, potentially indicating radical removal of malignant cells. The levels of FASLG in patients who later died or had a relapse of NSCLC were lower at all three timepoints compared to surviving patients without relapse, indicating that FASLG may be used as a prognostic biomarker. The biomarkers were confirmed using microarray data. In conclusion, quantitative proteomics could be used for NSCLC identification but may also provide information on radical surgical removal of NSCLC and post-surgical prognosis.

## 1. Introduction

Lung cancer is the number one cause of cancer-related deaths globally, causing around 1.8 million deaths annually [1,2]. Non-small cell lung cancer (NSCLC) accounts for approximately 85% of lung cancer cases, with lung adenocarcinoma (LUAD) and squamous cell carcinoma of the lung (SCC) being the most common forms [3,4]. The aim of this study was to identify protein biomarkers in the plasma of patients with primary NSCLC and to evaluate their potential usefulness in diagnosing and evaluating surgical resection of NSCLC. In this study, the tumor, node, and metastasis (TNM) 7th edition for lung cancer were used [5]. The methods used in the clinic to detect, diagnose, histologically subtype, and monitor lung cancer are mainly chest X-ray, bronchoscopy, and biopsy. An X-ray has a relatively low sensitivity and small tumors or tumors that are overshadowed by boney structures run a high risk of evading detection [6]. Furthermore, repeated radiographies in the form of a chest X-ray, computed tomography (CT), and positron emission tomography (PET), in lung cancer patients lead to radiation exposure levels exceeding recommended rates [7]. Bronchoscopy is time-consuming, costly, and invasive and comes with a risk of complications [8,9,10].

Next generation sequencing (NGS) is commonly used for guiding the choice of post-surgical treatment. NGS allows detection of changes such as substitutions, indels and rearrangements in, for example, proto-oncogenes in the tumor tissue [11]. Surgical removal of the tumor mass is a widely used treatment option for the early stages of NSCLC and is the form of treatment with the highest success rate [12,13,14,15,16]. Treatment of NSCLC can induce remission, but the majority of patients experience relapse and disease progression [17,18]. Unfortunately, this high recurrence rate of NSCLC is also responsible for the high mortality rate [19,20]. The survival rates for lung cancer overall and, specifically for NSCLC, are low, ranging from 15–19% 1 year survival for stage IV to 81–85% 1 year survival for stage I [4,21,22,23]. Yet another hindrance to the long-term survival of NSCLC patients is failure to diagnose the cancer at an early stage [1,24]. Earlier diagnosis leads to better survival rates, fewer treatment-associated comorbidities, lower health care costs, and early identification. Surgical removal of low stage NSCLC has been shown to generate 5-year survival rates as high as 70% [1,21,25]. Despite this, screening of at-risk populations for lung cancer is only commonplace in certain parts of the world [26,27]. The Dutch–Belgian Randomized Lung Cancer Screening Trial (NELSON) study, a randomized controlled trial of current and former smokers using low dose radiation CT, without contrast, revealed increased numbers of non-symptomatic NSCLC patients in early stages eligible for surgery, along with lower mortality in the screened cohort [28]. The national lung screening trial (NLST) in Sweden also showed a significantly lower mortality among screened patients but was not able to show any differences between low-dose CT and conventional chest X-ray as a screening method [29].

The search for biomarkers in cancer is an ongoing hot topic. Proteins may potentially be used for monitoring, predicting prognosis, measuring response to treatment, and detecting relapse [30]. Unfortunately, there are few known prognostic biomarkers for lung cancer in clinical use [31,32]. Discovering candidate biomarkers in blood has emerged as an attractive alternative to conventional techniques due to its minimally invasive nature, it does not require elaborate preparation, and allows for repeated sampling with ease and minimal risk for the patient. In the current study, we explored potential biomarkers in blood drawn before and after surgical resection of NSCLC.

## 2. Materials and Methods

The current study is a clinical study, performed in accordance with the Declaration of Helsinki and approved by the local ethics committee (Dnr: 2017/519). All patients signed written and informed consent forms prior to enrollment. 

### 2.1. Study Population

**Figure 1:**
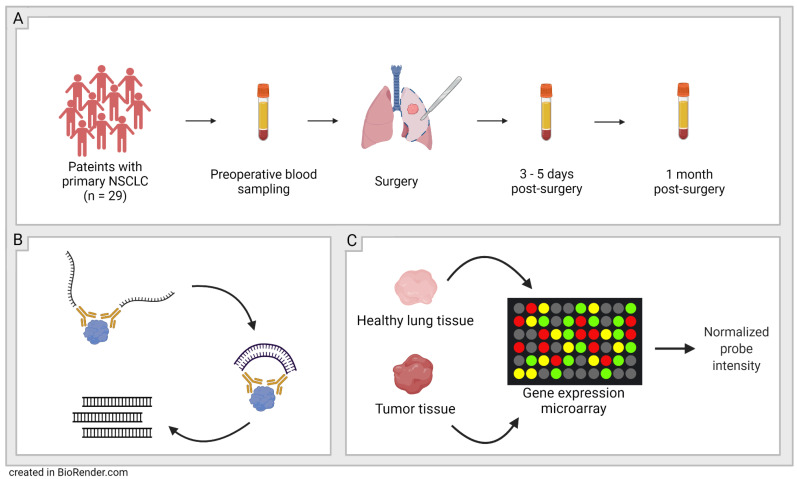
Schematic figure of study workflow. (**A**): Sampling-29 patients with primary non-small cell lung cancer (NSCLC) were included and blood plasma was sampled at three timepoints; before surgery, 3–5 days post-surgery, and 1 month post-surgery. (**B**): Olink’s proximity extension assay (PEA) technology was used to quantify the proteins in the plasma samples. (**C**): Validation-Protein expression patterns in plasma were validated with microarray data from NCBI’s GEO DataSets website.

A total of 29 patients undergoing surgery for resection of primary NSCLC (LUAD or SCC), stages IA–IIIA (T1a–T4, N0–N2, M0), according to the International Association for the Study of Lung Cancer’s (IASLC) TNM 7th edition, were included (Table 1) [5]. A total of 86 blood samples were collected; of these, 29 samples were preoperative, 28 were obtained 3–5 days post-surgery, and 29 were obtained 1 month after surgery (Figure 1). Timepoints for sampling were chosen based on the expected postoperative inflammation approximately 1 week post-surgery and the presumed downregulation of inflammation by 1 month post-surgery. It has previously been suggested that inflammation post-operation should be monitored for the first 4–7 days [33]. Exclusion criteria were chosen to minimize the risk of pathological processes other than NSCLC affecting the results of the proteomic analysis. Exclusion criteria include symptoms of ischemic heart disease, any unstable medical disorder, heart failure NYHA class III or IV, serum creatinine >140 µmol/L, diabetic subjects with glycated hemoglobin (HbA1c) > 48.0, as well as signs of liver cirrhosis, bleeding disorder or drug abuse. All patients were followed in regard to survival over 3.5 years after surgery.

### 2.2. Olink-Proximity Extension Assay (PEA)

A total of 92 proteins were analyzed using Olink’s Target 96 Oncology II panel (Olink, Uppsala, Sweden). The Target 96 Oncology II panel consists of pre-determined proteins. The panel was chosen based on proteins related to lung cancer. For more information on this panel, see the manufacturer’s webpage (https://www.olink.com/products-services/target/oncology-ii-panel/, accessed date on 27 August 2022).

The PEA analysis is a dual-recognition immunoassay that can be performed on very small plasma or serum samples down to 1.0 µL. The small amount of biospecimen needed is enough because of the exponential amplification that happens later on in the process. For every protein in the panel, there is a matched pair of antibodies that each carry a unique DNA tag (oligonucleotide). The oligonucleotides hybridize when brought into proximity due to the binding of the antibody pair to the same protein. Dual antibody binding is required which ensures a high specificity. Non-matched binding of antibodies to a protein does not yield a signal. The hybridized DNA tags include unique barcodes that can be detected by the system Fluidigm BioMark^TM^ HD standard real-time quantitative PCR. The oligonucleotides are then amplified in the presence of DNA polymerase, the number of cycles being determined by the protein concentration in each sample. Olink adds specially tailored blocking reagents to the analysis to reduce sample matrix interference. The qPCR is performed on eighty-eight customer samples and eight control samples that are assayed against the chosen panel of ninety-two proteins. This generates more than 8000 data points.

The PCR technique used by Olink allows for the readout of 96 protein assays in 96 samples simultaneously. The data are presented as normalized protein expression (NPX), a relative protein quantification unit on a log_2_ scale, for each protein biomarker in each sample. This allows for the identification of changes in individual protein levels across the sample set. A high NPX value equals a high protein concentration. Olink’s built-in quality control system uses three internal controls in each of the 96 wells of the sample plate. Additional sample controls for estimation of precision by intra- and inter-CVs (coefficients of variance), negative controls for the setting of the background levels for each protein, to calculate the limit of detection (LOD), and plate controls to compensate for potential variation between run plates are added.

Proteins with less than 15% detectability, i.e., proteins found in less than 15% of samples, according to Olink’s predetermined LOD were removed from the analysis. All 92 proteins in the Olink Target 96 Oncology II panel remained in the analysis. All samples were analyzed simultaneously. Further information on detection limits, assay characteristics, assay performance, and validation for each protein is available on the manufacturer’s webpage (http://www.olink.com, accessed date on 27 August 2022).

### 2.3. Confirmation of the Findings in Larger Cohorts

Microarray data from larger cohorts of subjects with NSCLC as well as healthy lung tissue were accessed through the NCBI’s GEO DataSets website (https://www.ncbi.nlm.nih.gov/gds, accessed date on 23 September 2022) (National Library of Medicine, Rockville Pike, Bethesda, MD, USA). In the current study dataset GEO: GSE10072 describing gene expression in NSCLC tumor tissue and healthy lung tissue from separate controls, and dataset GEO: GSE19804 describing paired NSCLC tumor tissue and adjacent healthy lung tissue were used to validate the patterns of protein expression found in plasma.

### 2.4. Statistical Analysis

Descriptive statistics are presented in the form of mean, range, subject number (*n*), and percentage of subjects. Statistical analyses were carried out by Olink through their offered statistical analysis services and in GraphPad Prism version 9.3.0 (GraphPad Software, San Diego, CA, USA). Olink analyses the data by fitting a linear mixed-effects regression model with each patient and cancer type considered as random effects. *p*-values are adjusted for multiple testing using the Benjamini–Hochberg approach with a false discovery rate (FDR) set to 0.05. Posthoc testing of the significant proteins is performed by calculating estimated marginal means, comparing the timepoints in a pairwise manner. *p*-values generated by the posthoc test were adjusted for multiple comparisons with Tukey’s method. Comparisons of smaller groups of samples were performed with the Mann–Whitney test. A cox proportional hazards model was performed in GraphPad Prism. Statistical significance was defined as **** (*p* < 0.0001), *** (*p* < 0.001), ** (*p* < 0.01), * (*p* < 0.05) and ns (*p* > 0.05).

## 3. Results

### 3.1. Proteomic Analysis

All 92 unique proteins in the Olink Target 96 Oncology II panel were detected in more than 75% of the samples. Of the 92 proteins, 63 (68%) were found to have a significant difference between the three timepoints after adjusting the *p*-values for multiple testing. The 12 proteins with the lowest adjusted overall *p*-values generated by a linear mixed-effects regression model were interleukin-6 (IL-6), mucin-16 (MUC-16), furin, protransforming growth factor alpha (TGFα), hepatocyte growth factor (HGF), vascular endothelial growth factor A (VEGFA), MHC class 1 polypeptide-related sequence A/B (MIC-A/B), amphiregulin (AREG), delta-like protein 1 (DLL1), tumor necrosis factor ligand superfamily member 6 (FASLG), transmembrane glycoprotein NMB (GPNMB), and tumor necrosis factor receptor superfamily member 6B (TNFRSF6B) (Table 2).

### 3.2. Comparing Three Timepoints: Pre-Op vs. 3–5 Days Post-Op vs. 1 Month Post-Op

Pairwise comparisons of preoperative, 3–5 days post-surgery, and 1 month post-surgery samples for every protein in the assay revealed significantly elevated plasma levels of six proteins (AREG, DLL1, furin, IL-6, TGFα, and TNFRSF6B) in both the 3–5 days post-surgery and the 1 month post-surgery samples compared to the preoperative samples. Furthermore, the plasma levels of the proteins MUC-16 and VEGFA were also elevated but did not reach significant levels compared to samples preoperatively vs. 3–5 days for MUC-16 and preoperatively vs. 1 month for VEGFA (Table 2).

**Figure 2:**
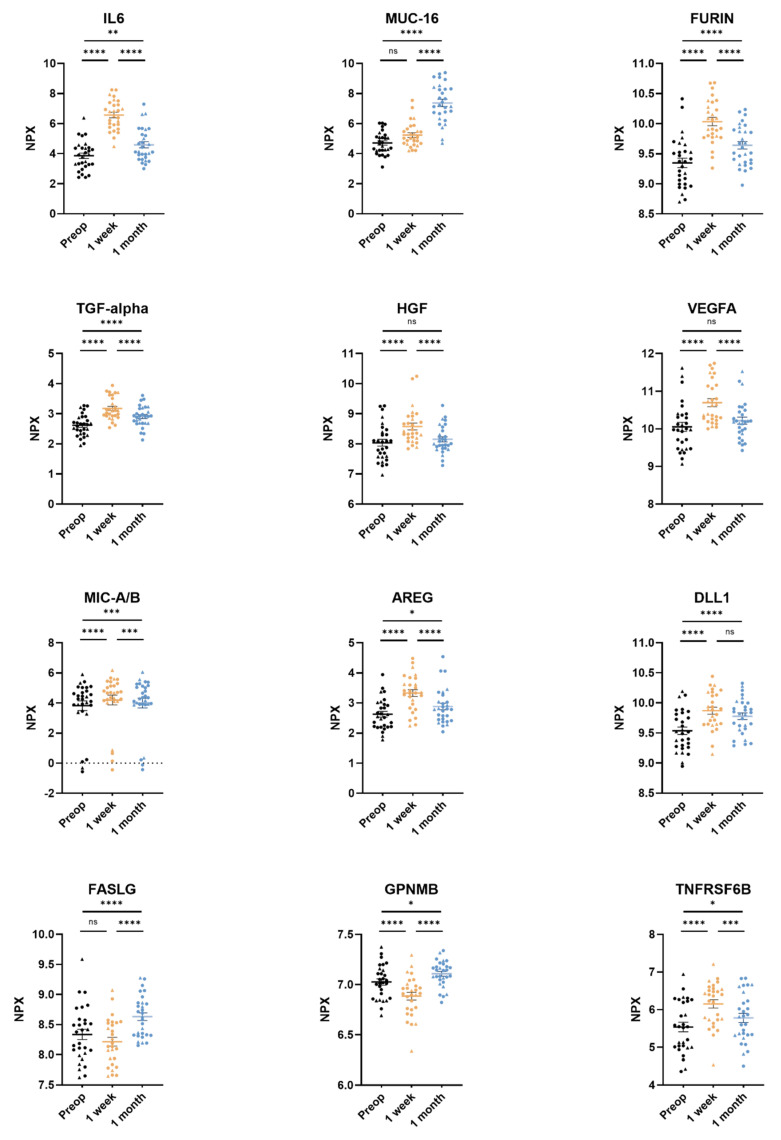
The twelve most significantly differing proteins in plasma from non-small cell lung cancer (NSCLC) patients. Adenocarcinoma datapoints are portrayed as circles and squamous cell carcinoma datapoints are portrayed as triangles. Plasma samples were taken preoperatively, 3–5 days post-surgery, and 1 month post-surgery. Protein levels pictured for interleukin-6 (IL6), mucin-16 (MUC-16), protein furin, transforming growth factor alpha (TGF-alpha), hepatocyte growth factor (HGF), vascular endothelial growth factor A (VEGFA), MHC class I polypeptide-related sequence A/B (MIC-A/B), amphiregulin (AREG), delta-like protein 1 (DLL1), tumor necrosis factor ligand superfamily member 6 (FASLG), transmembrane glycoprotein NMB (GPNMB), and tumor necrosis factor receptor superfamily member 6B (TNFRSF6B). Protein levels are expressed as normalized protein expression (NPX), a relative protein quantification unit on a log2 scale. Statistical significance was defined as **** (*p* < 0.0001), *** (*p* < 0.001), ** (*p* < 0.01), * (*p* < 0.05) and ns (*p* > 0.05).

The levels of MIC-A/B were significantly increased 3–5 days post-surgery compared to pre-operative levels (pre-op 3.82 ± 0.70 NPX, 3–5 days post-op 4.20 ± 1.69 NPX [*p* < 0.0001]) as well as levels 1 month post-surgery (pre-op 3.82 ± 0.70 NPX, 1 month post-op 4.00 ± 1.70 NPX [*p* = 0.0009]) (Figure 2, Table 2).

Plasma levels of FASLG were significantly higher at 1 month post-surgery compared to pre-operative levels (pre-op 8.34 ± 0.45 NPX, 1 month post-op 8.63 ± 0.34 NPX [p < 0.0001]) (Figure 2, Table 2). GPNMB followed the same pattern as FASLG (Figure 2, Table 2).

Plasma levels of HGF were significantly higher 3–5 days post-surgery compared to pre-operative levels (pre-op 8.04 ± 0.60 NPX, 3–5 days post-op 8.58 ± 0.58 NPX [*p* < 0.0001]) and decreased back to preoperative levels 1 month post-surgery (pre-op 8.04 ± 0.60 NPX, 1 month post-op 8.15 ± 0.45 NPX [*p* = 0.23]) (Figure 2, Table 2).

### 3.3. Comparison of Dead or Relapsed NSCLC to Survivors without Relapse

**Figure 3:**
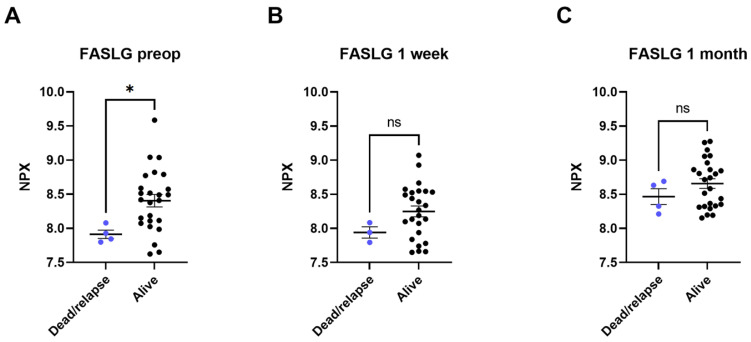
Plasma levels of tumor necrosis factor ligand superfamily member 6 (FASLG) compared between patients who died or had a relapse of cancer (dead/relapse) and patients still alive (alive). The comparisons were performed with Mann–Whitney tests. (**A**) comparison of preoperative samples, (**B**) comparison of 3–5 days post-surgical samples, (**C**) comparison of 1 month post-surgical samples. Protein levels are expressed as normalized protein expression (NPX), a relative protein quantification unit on a log2 scale. Statistical significance was defined as * (*p* < 0.05) and ns (*p* > 0.05).

Four of the twenty-nine patients included in this study died or had recurring NSCLC within the follow-up time of 3.5 years. In these patients, lower levels of FASLG were seen at all three timepoints compared to the survivors without relapse. The most significant difference was found between preoperative FASLG levels in patients who died or in patients with a relapse of NSCLC compared to survivors with no relapse (dead or relapsed patients’ pre-operative levels 7.91 ± 0.11 NPX, survivors’ pre-operative levels 8.40 ± 0.45 NPX [*p* < 0.05]) (Figure 3). A Cox Proportional-Hazards Model was performed and showed a parameter estimate for preoperative NPX-values of FASLG of -3.126. A negative parameter estimate indicates a decrease in the examined predictor variable (in this case NPX levels) which increases the hazard for the event (death or relapse) (*p*-value of 0.0672). Due to the low mortality and recurrence rate (*n* = 4), a bigger cohort might be needed to show such significance.

The association of survival and death or relapse was also examined for the two proteins HGF and MIC-A/B; however, no association could be found.

### 3.4. Validation Using GEO DataSets Microarray Data

Microarray data from two separate NSCLC cohorts deposited at the NCBI’s GEO DataSets website were used for validation of protein expression patterns found in plasma. One dataset describing gene expression in NSCLC tumor tissue and healthy lung tissue from controls (GEO: GSE10072) showed a significantly higher expression of MIC-A (GenBank NM_000247) in healthy control subjects compared to NSCLC (MIC-A control 8.18 ± 0.04, MIC-A NSCLC 8.00 ± 0.03 [*p* = 0.0044]). A second dataset describing gene expression in NSCLC tissue and adjacent healthy lung tissue (GEO: GSE19804) also showed a significantly higher expression of MIC-A (GenBank NM_000247) in healthy lung tissue compared to tumor tissue (MIC-A control 8.19 ± 0.05, MIC-A NSCLC 8.00 ± 0.06 [*p* = 0.0166]). Additionally, in this dataset, there was a significantly higher expression of FASLG (GenBank AF288573) in healthy tissue compared to tumor tissue (FASLG control 4.56 ± 0.05, FASLG NSCLC 4.42 ± 0.04 [*p* = 0.0239]). The proteins HGF and GPNMB were also investigated in the datasets, but the findings of this current study could not be validated. The unit of gene expression is normalized probe intensity (Table 3).

## 4. Discussion

Cancer is one of modern healthcare’s greatest challenges, causing millions of deaths every year. NSCLC is difficult to diagnose in its early stages and, once diagnosed, treatment of NSCLC is problematic as the disease often recurs even after initial remission [17,18]. Recently, Field et al. showed that lung cancer mortality was significantly reduced by low-dose CT screening [34]. Whilst screening does occur in many international centers, there is still a hesitation towards installing the practice, largely due to a missing organization of handling false-positive results. Screening using low-dose CT includes pre-scanning blood sampling and analyzing, as well as follow-up of kidney insufficiency, which requires additional setup and might therefore be challenging in some healthcare settings. Screening using blood samples would require less organization and would be more cost-effective than screening using low-dose CT and is therefore a highly promising field.

The present study explores the use of proteomics based on plasma to diagnose and predict the surgical outcome of NSCLC. In the current study, the patients included were sampled at three timepoints and served as their own controls. By omitting a separate control group, we minimize the risk of inherently different protein expression levels between individuals affecting the analyses. We used matched pair antibody-based PEA to analyze the incidence of 92 proteins within our patient cohort. The dual antibody binding of the method ensures a high specificity of the detected proteins, making the analytical method preferred over other antibody-based technologies that generate results in lower specificity due to the use of single antibody binding. In the present study, significant differences in plasma protein levels were found in 63 of the 92 analyzed proteins. Of those 63 proteins, the 12 proteins with the highest levels of significance were selected and presented separately.

MIC-A/B, which act as ligands to several immune cells including NK-cells, cytotoxic T-cells, and CD8^+^ T-cells, were found to be significant and among the 12 most significant proteins in the present study. MIC-A and MIC-B are expressed by many cancers, including NSCLC, and are involved in cell-mediated antitumoral responses [35,36]. Expression of tumor-related proteases has been shown to induce the shedding of MIC-A/B in some cancers, thereby allowing the malignant cells to evade cell-mediated antitumor immunity [35,36]. Furthermore, high expression of MIC-A/B has been found to be a positive prognostic factor in patients undergoing surgery for NSCLC, and a higher expression of MIC-A specifically has been associated with significantly longer survival times in NSCLC [37,38]. The significant increase of MIC-A/B levels after surgical removal of NSCLC in the present study indicates that the suppressant of the protein has been radically removed and MIC-A/B may therefore be used as an indicator of radical removal of NSCLC. This is further validated by the findings of the microarray data (Table 3) (Figure 2). In a current study by Djureinovic et al., the authors used Olink proteomics’ Oncology II panel to differentiate between NSCLC and different lung pathologies, both benign and malignant. In this study, MIC-A/B could not be used to differentiate between different lung pathologies. This is interesting but not entirely surprising as the disease areas studied are different in this publication and our current study [39].

FASLG, a member of the tumor necrosis factor ligand superfamily, was also found to be among the 12 most significant proteins in the present study. The binding of FAS to FASLG induces activation-induced cell death (AICD), cytotoxic T-cell- and NK-cell-induced cell death. The signaling pathway in which FASLG is active has a role in the apoptotic response of damaged cells, such as cancer cells [40]. The FASLG signaling pathway can be inhibited by the protein decoy receptor 3 (DcR3), which has been found to be elevated in lung and colon cancers [41,42]. DcR3 is often referred to as TNFRSF6B. TNFRSF6B is also to be found among the proteins in the Olink Target 96 Oncology II panel. Overexpression of DcR3 results in inhibited FASLG-induced cell death and cancerous cells evading the immune system [40]. In the current study, however, the plasma levels of TNFRSF6B were not higher prior to surgical treatment of NSCLC compared to samples taken at 3–5 days post-surgery and 1 month post-surgery. This could be explained by the fact that preoperative expression of TNFRSF6B was already higher than among healthy subjects, which would be in line with previous findings [41,42]. Moreover, recently Ali et al. showed that expression of FASLG is naturally higher in more differentiated, healthy tissues [43]. In the present study, plasma levels of FASLG were significantly higher 1 month after the surgical removal of NSCLC, potentially indicating that the NSCLC that had previously been suppressing the expression of FASLG has been radically removed (Figure 2). This is also validated by the patterns of gene expression in the accessed microarray data (Table 3). Interestingly, the levels of FASLG in plasma in patients who later died or had a relapse of NSCLC were lower at all three timepoints compared to the patients still alive with no relapse (Figure 3). This finding indicates that FASLG can be used as a prognostic biomarker for NSCLC as well as for the evaluation of radical surgical removal of NSCLC.

The protein HGF, a proto-oncogene that codes for a protein produced by fibroblasts in the lungs, stimulates cell motility, invasion, and morphogenesis, and acts as a potent mitogen for both healthy and cancerous cells in the bronchial epithelium [44]. Expression of HGF has been found to be elevated in tumor tissue of patients with NSCLC and especially in patients with tumor recurrence. Increased levels of HGF in plasma has been shown to correlate with poorer overall survival, and patients with stage I lung cancer with high levels of expressed HGF have a poorer prognosis than patients with stage II–III lung cancer with low expression of HGF [31]. Additionally, in a study by Masuya et al., it was shown that stromal expression of HGF in NSCLC cells correlated to a higher Ki-67 proliferation index, indicating a higher proliferation rate. It has also been shown that elevated expression of the HGF-receptor c-Met is associated with significantly lower survival [45]. In the present study, levels of HGF in plasma were initially significantly increased 3–5 days post-surgery but significantly lower between the 1 week and the 1 month timepoints, where the levels were again found to be in the same range as the pre-operative levels. Given the significant decrease in the relatively short follow-up time, a longer follow-up may have revealed a significant decrease over time compared to pre-operative levels of HGF among the surviving patients without relapse (Figure 2). In another publication using Olink’s PEA technology to study protein expression in cancerous cells in a fine-needle aspirate from NSCLC patients, HGF was among the top 49 abundant proteins and could be correlated to different stages of NSCLC, in line with the current study [46].

The glycoprotein NMB, or GPNMB, has been shown to be overly expressed in several human cancers, including NSCLC [47]. In a publication by Li et al., it was shown that overexpression of GPNMB has a role in the metastasis of cancerous tumors [48]. GPNMB is known to be expressed in monocyte-derived dendritic cells (Mo-DCs), where it is involved in inhibiting T-cell activation [49]. In the present study, plasma levels of GPNMB were significantly decreased 3–5 days post-surgery, and then significantly increased 1 month post-surgery (*p* < 0.0001 and *p* < 0.05, respectively). 

The plasma levels of AREG, DLL1, f”rin,’IL-6, MUC-16, TGFα, TNFRSF6B, and VEGFA were all significantly elevated after 3–5 days and 1 month aftersurgery. The increase may, in part, be explained by the inflammatory response caused by the surgical trauma itself. Due to surgical trauma, inflammatory cells including CD4^+^ T-cells are recruited, and bradykinin is released. Elevated levels of bradykinin may explain the increased levels of AREG [50,51]. Among the cytokines released due to surgical trauma and inflammation, IL-6 and TGFα are well characterized within the process of inflammation [52,53,54]. DLL1 is also known to play a central role in inflammation by increased production of IFN-γ and acting as a ligand in the NOTCH signaling pathway, which plays a role in regulating macrophage-mediated inflammation [55,56]. Angiogenesis driven by VEGFA is important in wound healing and is well known to be upregulated after surgery [57]. In tissue remodeling, such as in wound healing, expression of matrix metalloproteinases (MMPs) is enhanced by furin which is also in itself enhanced in immune cells, acting to attenuate the inflammatory response that follows surgical trauma [58,59,60]. MUC-16, a mucin naturally expressed in airway epithelium, functions to ensure both integrity and barrier function, thus contributing to the mucosal immune defense mechanism [61,62]. Thus, all of the aforementioned proteins (AREG, DLL1, furin, IL-6, MUC-16, TGFα, TNFRSF6B, and VEGFA) have previously been shown to have a connection to inflammation after surgery, which could explain the findings of the present study. Furthermore, proteins that have been shown to have an established connection to lung cancer show a significant change in protein expression levels after surgical removal of the tumor. A summary of the 12 proteins’ modes of action can be seen in Table 4.

To validate the patterns of protein expression found in plasma, the NCBI’s GEO DataSets website was searched for deposited microarray data. We accessed data from lung cancer biopsies and healthy lung tissue and were able to validate MIC-A and FASLG. We accessed two datasets, in both of which the expression of MIC-A was significantly higher in healthy lung tissue compared to tumor tissue. In one of the datasets, the expression of FASLG was also significantly higher in the controls compared to the tumor tissue. These results support the findings of the current study and encourage the status of these two proteins as potential biomarkers for diagnosing and predicting the outcome of NSCLC.

### Limitations

Proteins found in plasma are not necessarily specific to the lung and might reflect other processes such as malignancies and inflammation in other parts of the body. Given the inflammatory response secondary to the surgical trauma itself, additional samples at later timepoints would be preferable, for example at six, twelve, and eighteen months after surgery, since they could potentially reveal additional biomarkers related to NSCLC. Because of the number of biomarker candidates, it is perhaps more realistic to envision that characterization of NSCLC would take the shape of identifying a protein pattern to use as a biomarker rather than the discovery of one single protein [24]. One of the datasets used for the validation of this study’s findings consists of tumor tissue and healthy lung tissue from the same subjects. The use of a matched cohort increases the risk of selection bias which could potentially affect the differences in protein expression levels between tumor tissue and healthy tissue as the tissues are matched and the healthy lung tissue thus still originates from a patient with NSCLC.

## 5. Conclusions

Quantitative proteomics offers information on molecular interactions, signaling pathways, and biomarker identification by providing relative protein abundance. Using plasma as a proteomic source from patients with NSCLC, the present study implies that MIC-A/B, FASLG, and HGF are all valuable biomarkers and may not only be used as markers for radical removal of NSCLC but also to predict outcomes.

## Figures and Tables

**Table 1:** Patient characteristics. Descriptive statistics are presented as mean, range, number of patients, and percentage. Total number of patients is *n* = 29. *WHO* = World Health Organization, *R0* = macroscopically and microscopically radical, *R1* = macroscopically but not microscopically radical.

	*n* = 29
Sex, *n* (%)	
Male	14 (48)
Female	15 (52)
Age, years	
Mean (range)	71 (46–84)
Mortality, *n* (%)	
Alive	26 (90)
Deceased	3 (10)
Time from diagnosis to death, days	
Mean (range)	603 (264–786)
Time from surgery to death, days	
Mean (range)	551 (211–736)
Comorbidities, *n* (%)	
None known	17 (59)
Coronary artery disease	2 (7)
Diabetes mellitus	3 (10)
Hypertension	10 (34)
Arrhythmias	1 (3)
WHO performance status prior to surgery, *n* (%)	
0	15 (52)
1	14 (48)
Smoking history, *n* (%)	
Current	4 (14)
Former (> 6 weeks)	21 (72)
Never	4 (14)
Histopathological classification, *n* (%)	
Adenocarcinoma	21 (72)
Squamous cell carcinoma	8 (28)
Tumor stage, *n* (%)	
IA	13 (45)
IB	8 (28)
IIA	3 (10)
IIB	2 (7)
IIIA	3 (10)
Lung resection, *n* (%)	
Wedge resection	2 (7)
Segmental resection	2 (7)
Lobectomy	23 (79)
Bilobectomy	1 (3)
Pneumonectomy	1 (3)
Radicality, *n* (%)	
R0	26 (90)
R1	3 (10)
Neoadjuvant therapy, *n* (%)	
Combined chemotherapy and radiotherapy	2 (7)
Adjuvant therapy, *n* (%)	
Single therapy, chemotherapy	6 (21)
Combined chemotherapy and radiotherapy	1 (3)

**Table 2:** Protein levels of the twelve proteins with the lowest overall *p*-values. Protein levels preoperatively, 1 week post-surgery (3–5 days post-surgery), and 1 month post-surgery. Protein levels are expressed as normalized protein expression (NPX), a relative protein quantification unit on a log_2_ scale. Statistical significance is listed in the table, ns was defined as (*p* > 0.05).

Protein Abbreviation	Protein	NPX Preop (mean ± SD)	NPX 1 Week (mean ± SD)	NPX 1 Month (mean ± SD)	*p*-Value Preop vs. 1 Week	*p*-Value Preop vs. 1 Month	*p*-Value 1 Week vs. 1 Month
AREG	Amphiregulin	2.63 ± 0.51	3.33 ± 0.59	2.88 ± 0.58	*p* < 0.0001	*p* = 0.0237	*p* < 0.0001
DLL1	Delta-like protein 1	9.54 ± 0.33	9.87 ± 0.31	9.78 ± 0.29	*p* < 0.0001	*p* < 0.0001	ns
Furin	Protein furin	9.35 ± 0.41	10.03 ± 0.36	9.64 ± 0.33	*p* < 0.0001	*p* < 0.0001	*p* < 0.0001
IL-6	Interleukin-6	3.86 ± 0.95	6.57 ± 0.97	4.59 ± 1.09	*p* < 0.0001	*p* = 0.0016	*p* < 0.0001
MIC-A/B	MHC class 1 polypeptide-related sequence A/B	3.82 ± 0.70	4.20 ± 1.69	4.00 ± 1.70	*p* < 0.0001	*p* = 0.0009	*p* = 0.0003
MUC-16	Mucin-16	4.71 ± 0.74	5.24 ± 0.81	7.37 ± 1.26	ns	*p* < 0.0001	*p* < 0.0001
TGFα	Transforming growth factor alpha	2.62 ± 0.35	3.18 ± 0.37	2.91 ± 0.34	*p* < 0.0001	*p* < 0.0001	*p* < 0.0001
TNFRSF6B	Tumor necrosis factor receptor superfamily member 6B	5.54 ± 0.67	6.15 ± 0.57	5.78 ± 0.64	*p* < 0.0001	*p* = 0.0214	*p* = 0.0003
VEGFA	Vascular endothelial growth factor A	10.05 ± 0.63	10.69 ± 0.55	10.22 ± 0.49	*p* < 0.0001	ns	*p* < 0.0001
FASLG	Tumor necrosis factor ligand superfamily member 6	8.34 ± 0.45	8.22 ± 0.38	8.63 ± 0.34	ns	*p* < 0.0001	*p* < 0.0001
GPNMB	Transmembrane glycoprotein NMB	7.03 ± 0.17	6.89 ± 0.20	7.11 ± 0.13	*p* < 0.0001	*p* = 0.0207	*p* < 0.0001
HGF	Hepatocyte growth factor	8.04 ± 0.60	8.58 ± 0.58	8.15 ± 0.45	*p* < 0.0001	ns	*p* < 0.0001

**Table 3:** Gene expression of proteins MIC-A and FASLG according to separate cohorts accessed through the NCBI’s GEO DataSets website. Dataset GEO: GSE10072 comparing tissue from patients with NSCLC (*n* = 58) and healthy control patients (*n* = 49). Dataset GEO: GSE19804 comparing tissue from NSCLC (*n* = 60) and adjacent healthy lung tissue (*n* = 60). Gene expression was calculated by microarray techniques and expressed as normalized probe intensity.

Protein	GenBank	GEO	Gene Expression Cancer	Gene Expression Control	Significance
MIC-A	NM_000247	GSE10072	8.00 ± 0.03	8.18 ± 0.04	*p* = 0.0044
MIC-A	NM_000247	GSE19804	8.00 ± 0.06	8.19 ± 0.05	*p* = 0.0166
FASLG	AF288573	GSE19804	4.42 ± 0.04	4.56 ± 0.05	*p* = 0.0239

**Table 4:** Description of the top 12 proteins with the lowest *p*-values modes of action.

Protein Abbreviation	Protein	Mode of Action
AREG	Amphiregulin	Cytokine in the epidermal growth factor family. Binds to epidermal growth factor receptors and activates signaling in inflammatory processes, cell metabolism, and the cell cycle. Produced by immune cells [63].
DLL1	Delta-like protein 1	A NOTCH ligand. Regulates immune cells. Released from T-cells and eosinophilic cells. Secretion is enhanced by interleukin 1β. Has a positive correlation to systemic inflammation [64].
Furin	Protein furin	Cleaves and activates matrix metalloproteases, integrins, and cadherins (cell adhesion molecules). Expression is upregulated by tissue hypoxia [65].
IL-6	Interleukin-6	Involved in B-cell stimulation and induction of hepatic acute phase proteins. Increases thousand-fold in blood during inflammation. Signaling is dominated by signal transducer and activator of transcription 3 (STAT3) activation [66].
MIC-A/B	MHC class 1 polypeptide-related sequence A/B	Cancer cell-surface molecules. Activates cytolytic properties in natural killer cells and cytotoxic T-cells. Shedding of MIC-A/B by cancer cells leads to their escape from cell-mediated antitumor immunity [37].
MUC-16	Mucin-16	Glycoprotein is expressed by epithelial cells. Major component of mucus providing hydration and lubrication. Regulates mucosal defense of epithelial cells [61].
TGFα	Transforming growth factor alpha	Expressed by wound macrophages. Mediates angiogenesis, epidermal regrowth, and formation of granulation tissue [67].
TNFRSF6B	Tumor necrosis factor receptor superfamily member 6B	A soluble receptor also known as DcR3. Inhibits FASLG-induced cell death which potentially leads to the survival of malignant cells [42].
VEGFA	Vascular endothelial growth factor A	Induces angiogenesis and is important in wound healing. Plasma levels have been proven to rise post-surgery corresponding to the extent of the operative intervention [57].
FASLG	Tumor necrosis factor ligand superfamily member 6	Produced by activated T-cells and natural killer cells. Induces cell death of damaged cells. Naturally higher expression in healthy tissue. The binding of DcR3 to FASLG inhibits its function [40].
GPNMB	Transmembrane glycoprotein NMB	A transmembrane protein expressed by monocytic dendritic cells. Can inhibit T-cell activation [49]. Involved in metastasis of small-cell lung cancer [48].
HGF	Hepatocyte growth factor	A proto-oncogene, the protein stimulates cell motility, invasion, and morphogenesis. Acts as a potent mitogen [44]. High expression in NSCLC correlates with poor overall survival [31].

## Data Availability

Data is available on request from the corresponding author. Publicly available datasets were analyzed in this study. This data can be found here: [https://www.ncbi.nlm.nih.gov/gds, accessed on 23 September 2022].
